# Examining Nurses’ Views on Positive Clinical Working Environments Integrating With Their Religious Beliefs and Determining Their Metaphors: Qualitative Research

**DOI:** 10.1155/jonm/4498087

**Published:** 2025-12-12

**Authors:** Serpil Özcan, Gülcan Bahçecioğlu Turan

**Affiliations:** ^1^ Department of Fundamental of Nursing, Ataturk University, Erzurum, Turkey, atauni.edu.tr; ^2^ Department of Fundamental of Nursing, Faculty of Nursing, Ataturk University, Erzurum, Turkey, atauni.edu.tr

**Keywords:** nurses, religious beliefs, work conditions, workplace

## Abstract

**Objective:**

This study aims to examine nurses’ views on positive clinical work environments integrated with their religious beliefs and to determine their metaphors.

**Method:**

The study population consisted of nurses working in a university hospital in eastern Turkey between May 6 and 16, 2025. The study sample consisted of 41 nurses who agreed to participate. The criterion sampling method was used. The research data were collected using the “Introductory Information Form” and “Semistructured Questionnaire.” Content analysis was used to evaluate qualitative data, and descriptive statistical methods (frequency, percentage, and mean) were used to evaluate quantitative data. This study followed the COREQ checklist.

**Results:**

The analysis of data obtained from interviews with nurses resulted in the creation of 3 themes and 18 codes: “The Impact of Religious Beliefs on Professional and Team Relationships,” “The Impact of Religious Beliefs on Coping with Clinical Challenges,” and “The Impact of Religious Beliefs on a Positive Clinical Environment.” The nurses’ metaphors regarding their religious beliefs and their profession were divided into three different categories based on their common characteristics. Twenty‐three different metaphors were created for these categories.

**Conclusion:**

This study highlights, for the first time, how nurses’ religious beliefs shape their perceptions of a positive clinical work environment. The findings demonstrate that these beliefs influence professional relationships, coping strategies, and the overall atmosphere of the workplace. In addition to contributing to the literature, the results have managerial relevance: acknowledging and respecting employees’ individual values can foster motivation, cooperation, and resilience, while also enhancing job satisfaction and the quality of patient care.

## 1. Introduction

Nursing is an applied discipline that combines professional knowledge, ethical sensitivity, and human values. In this discipline, which is responsible for providing care at its core, the physical needs of individuals and their psychological, social, and spiritual aspects are approached from a holistic perspective. Within this holistic understanding, the nurse’s personal values, worldview, and belief system are directly or indirectly reflected in the care process. In particular, religious beliefs influence how nurses perceive their professional roles, motivate their practice, and help them cope with clinical challenges [1, 2].

Religious beliefs may serve as an internal stabilizing factor in stressful and emotionally intense environments and can foster empathy and understanding in the care process [1] and can also foster empathy and understanding in the care process [3]. Faith‐based values provide a conscientious reference point in nurses’ decision‐making [4], influence the provision of health services [5, 6], and contribute to professional orientation and organizational commitment [7].

Religious and spiritual beliefs also contribute to the overall climate of the work environment by helping individuals gain a sense of meaning and purpose, thus enhancing emotional balance and psychological resilience [8, 9]. According to a study, strong religious beliefs of nurses increase all dimensions of organizational commitment, which positively impacts organizational performance [10].

Positive working environments in nursing are characterized by peace, high job satisfaction, low stress levels, and improved care quality [11, 12]. In this context, considering the positive influence of religious beliefs on work environments, it is essential to conduct in‐depth research on this topic. Particularly in eastern Turkey, where religious traditions and collective values strongly shape sociocultural life, examining the interaction between nurses’ belief systems and their professional environments offers unique insights that may not be captured in other contexts. However, despite the growing recognition of spirituality in nursing, existing studies often remain descriptive and do not critically explore how religious beliefs intersect with organizational culture, team dynamics, and care outcomes. This gap highlights the need for research that not only documents these influences but also analyzes their implications for creating positive and sustainable clinical environments.

Based on this need, it is thought that examining nurses’ views and metaphors on this subject with a qualitative research method will be effective in obtaining more in‐depth and context‐specific data. For this reason, this study aims to examine nurses’ views on positive clinical work environments that integrate with their religious beliefs and to determine their metaphors. The research seeks answers to the following questions.

1. What are nurses’ views on the effects of their religious beliefs on clinical work environments?
2. What are nurses’ religious beliefs metaphors for their profession?
3. What are nurses’ metaphors of a positive clinical environment?

## 2. Methods

### 2.1. Type of Research

This research used a descriptive phenomenology design and metaphor analysis technique based on the phenomenological method. “Metaphor analysis” is a technique based on the phenomenological research method within qualitative research methods. Metaphor analysis is a research method used to explore abstract and complex concepts by drawing analogies with familiar ones [13]. Descriptive phenomenology was chosen to capture and describe nurses’ lived experiences without aiming to build a theory. Grounded theory and other approaches were not applied, as the study sought rich descriptive insights rather than theoretical model development.

### 2.2. Study Population and Sample Selection

The population of the study consisted of nurses working in a university hospital in eastern Turkey between May 6 and 16, 2025. The study sample consisted of 41 nurses who agreed to participate. The criterion sampling method was used. In this context, nurses with at least 1 year or more of working experience were included in the sample. The sample size of 41 nurses was deemed sufficient for qualitative inquiry, and the study was completed at this point because data saturation had been reached. Saturation was determined when no new codes or themes emerged from the last three consecutive interviews and the incoming data began to repeat previously defined patterns. This number also reflects criterion‐based purposive sampling of nurses with clinical experience, which strengthened the validity and credibility of the sample. The reason for choosing this period is that the nurses have reached the experiential accumulation to observe and evaluate the dynamics of the clinical environment adequately and to make sense of how their religious beliefs interact with professional practice and a positive working environment.

### 2.3. Data Collection Tools and Features

A Descriptive Information Form consisting of six questions (age, gender, marital status, educational status, professional experience, and unit of employment) and five semistructured questions and two metaphor questions created by the researchers with the support of the literature were used to collect the research data. The semistructured questions and metaphor questions were submitted to six expert opinions. After receiving expert opinions, three pilot applications were conducted (pilot applications were not included in the analysis), and the semistructured interview form and metaphor questions were finalized. Each pilot interview lasted approximately 10 min and was conducted with nurses meeting the inclusion criteria. Feedback from these pilots indicated that some questions were perceived as overlapping or unclear. Based on this feedback, minor wording adjustments were made to enhance clarity, and redundant probes were removed. This process ensured that the final instrument was both comprehensible and contextually relevant. Semistructured questions and metaphorical questions are given in the following.

1. How would you describe the impact of your religious beliefs on your nursing profession?
2. What does a positive clinical work environment mean to you?
3. How do your religious beliefs provide you with direction in the face of challenges you face in the clinical setting?
4. How do you think your beliefs play a role in your relationships with your teammates in the clinic?
5. How can your religious beliefs contribute to a positive working environment?

#### 2.3.1. Metaphor Questions

- Religious beliefs are like a “…” for me in the nursing profession. Because…
- A positive clinical environment is like a “…” in my eyes. Because…

### 2.4. Data Collection

The research data were collected from 41 nurses working in a university hospital in eastern Turkey between May 6 and 16, 2025. After the participants were informed about the purpose and method of the study, the data were collected by the nurse researcher, an academician (G.B.T.) trained in qualitative research based on the principle of volunteerism and to prevent bias.

Data collection started when the nurses were available without interfering with their workflow in the clinic. The interviews were conducted face‐to‐face after determining the day and time when the nurses were available. The interviews were conducted in the nurses’ lounge in the clinics where the nurses worked. Only the researcher and the participant were present during the interviews. Each nurse was given a code name for easy analysis without using their names (V1, V2, …, V41). Before starting the interviews, information about the purpose of the study and the data collection method was given. Then, after obtaining the written/verbal consent of the participants, the introductory information form, semistructured questions, and metaphor questions were directed to the participants, and their answers to the questions were recorded on the voice recorder. The interviews lasted 10–15 min.

#### 2.4.1. Data Evaluation

The study’s quantitative data were analyzed using the SPSS 23 package program. Frequency, percentage, mean, and standard deviation values were used to evaluate the data related to descriptive characteristics.

Qualitative data were analyzed under the guidance of the Consolidated Criteria for Reporting Qualitative Research (COREQ) checklist, which was prepared for reporting qualitative research after the research design was decided [14].

After the in‐depth interviews were completed, the interviews recorded on the voice recorder were transferred to the computer with code names. They were then transcribed into written text by the researchers. The data from the semistructured questions were analyzed using the content analysis (inductive) method. The data were analyzed by hand coding separately by two researchers (G.B.T. and S.Ö.) trained in qualitative research. In terms of reflexivity, researchers acknowledged that their own professional and cultural backgrounds, including personal values and beliefs, could potentially shape coding and interpretation. To minimize this influence, coding was conducted independently by two researchers and subsequently compared for consistency. Reflexive notes were taken throughout the analysis to ensure that interpretations were based on participants’ narratives rather than researchers’ assumptions. This process enhanced the reliability and credibility of the findings. Then, the analyses made by both researchers were compared and finalized. The content analysis method was carried out in four stages. Then, the analyses made by both researchers were compared and finalized. The content analysis method was carried out in four stages [15]:

 Stage 1: Coding the data: the information gathered from each interview question was analyzed and segmented into parts that formed a meaningful whole, from which codes were individually generated.
 Stage 2: Creation of themes: the generated codes were analyzed collectively, with an effort to identify commonalities among them. Based on their scope and depth, the codes were categorized, and themes were developed accordingly.
 Stage 3: Structuring the data based on codes and themes: the data were systematically organized to facilitate defining and interpreting them in relation to specific phenomena. These definitions were then presented within the findings.
 Stage 4: Findings‐based interpretation: following the presentation of the data with percentages, each finding was defined, and the researcher’s interpretations and reflections were provided accordingly.

The metaphor questions of the study were analyzed separately by both researchers using the critical metaphor analysis (CMA) method developed by Charteris‐Black [16], and the results were compared and finalized. Critical Metaphor Analysis was conducted in three stages [16].

1. Defining metaphors
2. Interpretation of metaphors
3. Explanation of metaphors

The use of metaphor analysis was justified by its ability to capture participants’ implicit perceptions and abstract experiences in ways that conventional phenomenological coding alone may not reveal. While descriptive phenomenology identifies lived experiences, metaphor analysis adds value by uncovering symbolic meanings and shared cultural representations, thereby providing a richer and more nuanced understanding of how nurses conceptualize religious beliefs and positive clinical environments.

### 2.5. Limitations of the Study

This study was conducted with nurses working in a university hospital in eastern Turkey over a specific period, and the findings obtained are limited to the experiences and perceptions of nurses in this institution. Therefore, the study’s results cannot be generalized to nurses working in different institutions.

### 2.6. Research Ethics

Ethics committee permission (numbered 2025/06‐34) was obtained from Firat University Noninterventional Research Ethics Committee for the implementation of the study. Institutional permission was obtained from the institution where the research would be conducted. Participants were informed that the data would be used solely for research purposes and that they had the right to withdraw at any time. Before starting the research questions in face‐to‐face interviews, written/verbal consent was obtained from the participants, who voluntarily agreed to participate. The research was conducted following the Declaration of Helsinki.

## 3. Results

The religious beliefs of the nurses participating in the study, their views on a positive clinical work environment, and their metaphors were analyzed. The mean age of the nurses participating in the study was 30.34 ± 5.73 years. Among the nurses, 82.9% were female, 53.7% were single, 61% were graduates, 43.9% had 1–5 years of professional experience, and 63.4% worked in a specialized unit (Table 1).

**Table 1:** Demographic characteristics of nurses.

Characteristics	Variables	*n* (%)
Gender	Female	34 (82.9)
	Male	7 (17.1)
Marital status	Single	22 (53.7)
	Married	19 (46.3)
Education status	Graduate	25 (61)
	Postgraduate	16 (39)
Professional experience	1–5 years	18 (43.9)
	6–10 years	16 (39)
	11 years and over	7 (17.1)
Working unit	Internal unit	8 (19.5)
	Surgical unit	7 (17.1)
	Specialised unit	26 (63.4)
Total		41 (100)
Age		***X*** ± **SD (min**–**max)**
		30.34 ± 5.73 (23–48)

As a result of the analysis of the data obtained from the interviews with the nurses, 3 themes and 18 codes were formed as “The Effect of Religious Beliefs on Professional and Team Relationships,” “The Effect of Religious Beliefs on Coping with Clinical Difficulties” and “The Effect of Religious Beliefs on Positive Clinical Environment” (Table 2).

**Table 2:** Themes and codes obtained from interviews with nurses.

Theme	Code	Volunteer	n
The effect of religious beliefs on professional and team relations	Being patient/compassionate	1, 3, 5, 6, 7, 10, 11, 12, 13, 15, 16, 18, 20, 22, 24, 26, 30, 31, 34, 35, 36, 37, 38, 39, 40	25
	Being respectful/understanding	1, 2, 4, 5, 6, 7, 10, 11, 14, 15, 16, 17, 20, 26, 27, 28, 29, 30, 31, 33, 34, 35	22
	Awareness of professional/ethical responsibility	4, 5, 7, 10, 13, 14, 15, 16, 18, 19, 21, 25, 26, 27, 31, 35, 41	17
	Fairness	3, 6, 10, 13, 14, 15, 16, 19, 21, 22, 26, 29, 36, 38	14
	Positive perspective	1, 2, 18, 20, 23, 27, 28, 30, 33, 39	10
	Provides stress management	6, 7, 10, 15, 28, 31, 34, 38, 40	9
	Supports collaboration	5, 10, 14, 26, 30, 31, 38	7
	Provides the ability to empathise	6, 20, 26, 30, 35, 36, 41	7
The effect of religious beliefs on coping with clinical difficulties	Ability to remain patient/calm	1, 3, 4, 5, 6, 9, 10, 11, 12, 13, 14, 20, 21, 22, 24, 30, 34, 35, 39, 41	20
	Trust in God	2, 5, 7, 10, 15, 16, 20, 24, 25, 26, 28, 30, 35, 40	14
	Moral behavior	3, 8, 14, 17, 22, 27, 33	7
The effect of religious beliefs on a positive clinical environment	Peaceful/happy working environment	3, 4, 5, 9, 10, 12, 13, 14, 15, 18, 19, 20, 27, 30, 31, 32, 33, 34, 35, 36, 37, 38, 40, 41	24
	Harmonious/supportive working environment	7, 8, 10, 11, 12, 16, 18, 22, 23, 24, 26, 27, 28, 30, 31, 37, 38, 39, 40, 41	20
	Increases productivity/motivation	6, 7, 9, 10, 12, 16, 18, 20, 22, 24, 25, 28, 29, 31, 33, 36, 41	17
	Provides tolerance	5, 10, 13, 15, 18, 26, 27, 30, 31, 38, 41	11
	Provides patient/employee satisfaction	6, 13, 14, 15, 16, 20, 25, 28, 30, 31, 32	11
	Encouraging patient care	2, 7, 13, 22, 27, 30, 31, 34, 36, 38	10

### 3.1. Theme 1: The Effect of Religious Beliefs on Professional and Team Relations

For Theme 1, 8 codes were identified as “being patient/compassionate,” “professional/ethical responsibility awareness,” “being respectful/understanding,” “being fair,” “positive perspective,” “provides stress management,” “supports cooperation,” and “provides empathy.” Most of the nurses stated that their religious beliefs were very effective in their feelings of patience and compassion toward their patients and teammates. In addition, nurses indicated that they were more aware of their professional and ethical responsibilities thanks to their religious beliefs, which caused them to act justly, and that being respectful and understanding toward people was a requirement of their religious belief. In addition, the nurses stated that religious beliefs facilitated coping with professional stress, encouraged them to cooperate with their teammates, and contributed to their more empathetic approach to patients (Table 2). The following are sample interview excerpts related to the codes identified under Theme 1:

V6: “My religious belief enables me to be more compassionate toward patients, and I can be more empathetic toward patients and their relatives” (female, married, surgical unit).

V10: “According to my belief, patience and acting in accordance with human dignity are valuable. For this reason, it enables me to fulfil my professional responsibilities better and guides me in my ethical and moral stance” (female, surgical unit).

### 3.2. Theme 2: The Effect of Religious Beliefs on Coping With Clinical Difficulties

Three codes were identified for Theme 2: “remaining patient/calm,” “trusting,” and “acting morally.” The nurses stated that their religious beliefs enabled them to remain more calm and patient in the face of difficulties in the clinical environment with the power given by their religious beliefs, to believe that everything comes from God and has a reason, and to exhibit a more moral stance in the face of difficulties (Table 2). The following are sample interview excerpts related to the codes identified under Theme 2:

V1: “It makes me more patient and calm in the face of difficulties” *(female, surgical unit*).

V15: “I draw strength from my religious belief when coping with stressful situations. Prayer, patience, and trust…” *(female, internal unit*).

### 3.3. Theme 3: The Effect of Religious Beliefs on a Positive Clinical Environment

For Theme 3, six codes were identified as “peaceful/happy working environment,” “harmonious/supportive working environment,” “increases productivity/motivation,” “provides tolerance,” “provides patient/employee satisfaction,” and “encourages patient care.” Nurses provided important views that their religious beliefs effectively provided a positive clinical environment. The nurses stated that they endeavored to create a peaceful and happy working environment with the teachings of their religious beliefs and provided a harmonious and supportive working environment. They noted that this peaceful and happy clinical environment increased their productivity, motivation, and encouraged them to provide patient care. In addition, nurses stated that in this positive clinical environment provided by religious beliefs, tolerance prevailed and patient and staff satisfaction increased (Table 2). The following are sample interview excerpts related to the codes identified under Theme 3:

V13: “In the peaceful environment provided by religious belief, I approach patients with more patience and compassion. I work happier, and this reflects positively on patient care” *(female, internal unit*).

V27: “Religious beliefs provide an environment of tolerance in the clinic” *(29 years old, male, married, graduate, 6-10 years, specialized unit*).

**Table 3:** Religious belief metaphors about nursing identity and practice.

Category	Metaphor	*n*	
The contribution of belief to ethics and a humanitarian approach	Guide(V4,V11,V12,V14,V22,V33,V35), compass (V1,V30,V36,V38,V41), indispensable (V3,V26), compassion (V19,V21), touchstone (V27,V28), safe house (V5), light (V6), chance (V13), award (V17), life (V18), sun (V20), *Added value* (V23), contribution (V24), *Guardian angel* (V31), principle (V32), peace (V34), important (V39)	17	Nurses described their religious beliefs as moral compasses and guiding lights that shape their professional ethics and humanistic care. These metaphors highlight how belief directs nurses toward compassion, justice, and integrity in patient relationships.
Contribution to professional resilience and satisfaction	Guide (V7), compass (V10), armour (V15), armour (V16), root (V8), worship (V40)	6	Religious beliefs were viewed as internal sources of strength and endurance. Metaphors like *armour* and *root* represent protection and stability, while *worship* and *guide* express meaning, motivation, and devotion to the profession.
Perception of religious neutrality in profession	Unimportant (V2,V9,V25,V29), preference (V37)	2	A few nurses emphasized that nursing should remain neutral regarding religion. These metaphors reflect the view that professional practice must focus on patient care without religious influence or bias.

Note: V:volunteer; *n* = frequency column indicates the number of participants who used each metaphor. The total number of metaphors per category is also presented. This table presents metaphors that nurses used to describe how their religious beliefs influence their professional identity and nursing practice.

Nurses’ religious beliefs and metaphors for their profession were grouped into three different categories according to their common characteristics. The categories were determined as “contribution of belief to ethical and humanitarian approach,” “contribution to professional resilience and satisfaction,” and “perception of religious neutrality in the profession.” It was determined that some of the metaphor types produced by nurses (guide and compass) were included in more than one category. The guide (*n* = 8) and compass (*n* = 6) metaphors, which were the most frequently produced metaphors, were included in two different categories (Table 3). The frequency of each metaphor, shown in the frequency (*n*) column, reflects the number of participants who used that expression. The metaphors “Guide” and “Compass” have been deliberately separated into two categories because they are used in different target areas and carry different contextual meanings. For example, the term “Guide” is used to define professional/ethical orientation under the theme of “professional and team relationships,” while it is used to express organizational orientation and unit management under the theme of “coping with clinical challenges/positive environment.” Similarly, “Compass” meant an internal ethical reference for nurses in one category, while in another category, it meant a reference to team/ward orientation under stress. Therefore, to preserve participants’ statements, coding was performed based on the meaning in context according to critical metaphor analysis, maintaining the same word from across categories.

Category 1: The Contribution of Belief to Ethics and Humanitarian Approach: in this category, nurses emphasized that their religious beliefs contribute to their ethical and humane approach to patients in their profession. In this category, 17 different metaphors were produced, and it was determined that “guide” and “compass” metaphors were used the most, with seven and five repetitions, respectively (Table 3). Sample statements regarding the metaphors created by nurses in Category 1 are given in the following:

V12: “Religious beliefs are like a guide for me in my profession because I approach my patients in line with my beliefs” *(female, surgical unit*).

V20: “My religious belief is like a sun for me in my profession because I can approach my patients more patiently and empathetically according to my religious belief” *(28 years old, female, single, graduate, 6*–*10 years, specialized unit*).

Category 2: Contribution to Professional Resilience and Satisfaction: within this category, nurses emphasized that their religious beliefs contributed to professional resilience and satisfaction. Within this category, six different metaphors were produced. Six different nurses produced metaphors in this category (Table 3). Sample statements regarding the metaphors created by nurses in Category 2 are given in the following:

V15: “I think religious beliefs are like armor in nursing because beliefs give the strength to cope with professional difficulties, increase intrinsic motivation and hope” *(female, internal unit*).

V40: “I think that religious beliefs are like worship in the profession because I feel the same peace and happiness while practicing my profession as I feel while practicing worship” *(female, internal unit*).

Category 3: Perception of Religious Neutrality in Profession: within the scope of this category, nurses emphasized that the nursing profession provides services without discriminating against religion, language, and race, and for this reason, religious beliefs should not affect the nursing profession. Two different metaphors were produced in this category. In this category, it was determined that the metaphor “it is unimportant” was used the most with four repetitions (Table 3). Sample statements regarding the metaphors created by nurses in Category 3 are given in the following:

V25: “I think it is insignificant because the dimension of belief in the profession does not affect the profession” *(male, specialized unit*).

V37: “It is a matter of choice. Because profession and religious belief should not be mixed” *(male, specialized unit*).

Nurses’ metaphors for a positive clinical environment were collected in three different categories according to their common characteristics. The categories were determined as “sense of peace and belonging in the clinical environment,” “spiritual well‐being and hope in the clinical environment,” and “perception of professional order and responsibility.” Nurses produced 24 different metaphors for the positive clinical environment. The most common metaphors were peace (*n* = 7), home (*n* = 7), and family (*n* = 4), as shown in the frequency (*n*) column of Table 4, which indicates the number of participants who expressed each metaphor.

**Table 4:** Metaphors about positive workplace environments.

Category	Metaphor	*n*	
Sense of peace and belonging in the clinical environment	Peace (V3,V4,V5,V15,V24,V34,V36), Home(V9,V11,V12,V25,V27,V30,V41), family (V10,V35,V37,V40), Paradise (V2,V28,V32), Source of happiness (V16,V33), Good work environment (V14,V26), equality (V1), being respectful (V1), Sea (V6), relief (V8), Source of value (V13), respect (V17), discipline (V18), sincerity (V22), advantage (V23), love the profession (V31), important (V39)	18	Nurses associate a positive clinical environment with feelings of peace, comfort, belonging, and harmony. Metaphors such as *home*, *family*, and *peace* symbolize emotional safety and mutual support, while *relief* and *source of value* reflect renewal and appreciation within the workplace.
Spiritual well‐being and hope in the clinical environment	Spring morning (V7), amusement park (V20), spring weather (V38)	3	These metaphors evoke vitality, freshness, and joy. Nurses view a positive work environment as spiritually uplifting and energizing, helping them start each day with renewed motivation and optimism
Perception of professional order and responsibility	Structured order/hierarchy (V19), justice (V21), Machine (V29)	3	Nurses perceive a well‐functioning clinical environment as organized, fair, and disciplined. The metaphors emphasize structure, teamwork, and equitable responsibility among staff, reflecting professionalism and system stability.

Note: V:volunteer; *n* = frequency column indicates the number of participants who used each metaphor. The total number of metaphors per category is also presented. This table presents metaphors that nurses used to describe the characteristics and emotional atmosphere of a positive clinical workplace.

Category 1: Sense of Peace and Belonging in the Clinical Environment: nurses emphasized that a positive clinical environment contributes to their sense of peace and belonging in the clinic and the profession. Within this category, 18 different metaphors were produced, and it was determined that “peace” and “home” metaphors were used the most, with seven repetitions (Table 4). Sample statements regarding the metaphors created by nurses in Category 1 are given in the following:

V34: “It is like peace. Because we are in the working environment more than at home. I work more efficiently in a peaceful environment” *(female, specialized unit*).

V41: “A positive clinical environment is like a home for me. Because I feel safe, peaceful, and supported there” *(female, internal unit*).

Category 2: Spiritual Well‐Being and Hope in the Clinical Environment: the nurses emphasized that the positive clinical environment provided them spiritual well‐being and hope. Within this category, three different metaphors were produced. Three different nurses produced metaphors in this category (Table 4). Sample statements regarding the metaphors created by nurses in Category 2 are given in the following:

V7: “I think it is a spring morning. Because it energizes and refreshes” *(female, specialized unit*).

V38: “In my eyes, it is like a spring morning. Because it refreshes one’s heart and makes the working environment pleasant” *(female, surgical unit*).

Category 3: Perception of Professional Order and Responsibility: nurses emphasized that a positive clinical environment contributes to professional order and responsibility. Three different metaphors were produced in this category. Three different nurses produced metaphors in this category (Table 4). Sample statements regarding the metaphors created by nurses in Category 2 are given in the following:

V19: “In my eyes, it is more like a political environment. I think we can be more productive when we work without internalizing the problems” *(female, featured unit*).

V29: “I think it is like a machine. Because a machine works in a very organized and orderly way. I liken clinical environments to that” *(male, specialized unit*).

Overall, metaphors related to the nursing profession (Table 3) primarily reflected moral guidance, ethical principles, and inner strength, while those describing the clinical environment (Table 4) highlighted belonging, harmony, and emotional peace. This distinction suggests that nurses view their faith as a personal guide in professional practice, whereas the workplace is perceived as a shared space of safety and support.

## 4. Discussion

This study aims to examine nurses’ views on positive clinical work environments that integrate with their religious beliefs and to determine their metaphors. Since there is no qualitative research on this subject, it is thought that the results of this study are striking and impressive. The findings highlight the ways in which nurses integrate their beliefs into professional practice.

In the study, nurses stated that their religious beliefs were most effective in being patient, having professional ethical responsibility, being respectful and understanding, being fair, providing a positive perspective, managing stress, being cooperative, and empathizing. According to Harris and Tao [9], religion/spirituality had a positive impact on spiritual well‐being, showed a negative correlation with emotional exhaustion and depersonalization, and was positively linked to personal achievement [9]. Gonçalves et al. [17], in their systematic review and meta‐analysis, concluded that interventions involving religion or spirituality are linked to improved mental health outcomes in both patient and healthy populations [17]. Seemann [2] reported that organizations affiliated with a particular religion attract employees with work ethics, colleagues are respectful, friendly, and helpful to each other and people are more altruistic, humble, and selfless, which is one of the most significant differences compared to nonfaith‐based organizations [2]. According to Turton and Francis [18], having a positive attitude toward prayer was linked to greater personal accomplishment and reduced levels of emotional exhaustion and depersonalization [18]. Habibian et al. [19] found that religious attitude was positively correlated with four dimensions of psychological well‐being: life purpose, self‐acceptance, personal growth, and environmental mastery [19]. In their systematic review, Perera et al. [11] stated that nurses worldwide engage in religious activities and spiritual behaviors to overcome stress and alleviate the negative consequences of work stress [11]. It has been reported in the literature that individuals with higher levels of belief maturity tend to be able to cope with stressors [20]. It was also found that religion and spirituality can contribute to job satisfaction among nursing professionals [11]. In this context, religion and spirituality reduce the harmful consequences of emotional labor, providing meaning, support, and an optimistic orientation toward life [11]. These findings suggest that religious beliefs influence professional practice and workplace culture by providing a shared moral framework, reinforcing ethical responsibility, and fostering trust and empathy among colleagues. In parallel with these findings, the metaphors such as “angel of goodness,” “peace,” “equality,” “kindness,” “compassion,” “sincerity,” “discipline,” “shelter,” and “light” used by the nurses in the research show that nurses find meaning in the workplace, develop resilience, and adopt a more optimistic attitude by feeding on religious/spiritual values.

In this study, nurses stated that religious beliefs were most effective in coping with clinical difficulties, mostly in trusting to remain calm and behaving morally. The nursing profession is considered an emotionally challenging profession due to heavy workload, shift work rotation, encountering the death of patients, encountering violence in the workplace, and many other work‐related stress factors. It often leads to various psychological difficulties and prolongs the level of stress. It is generally assumed that nurses are more prone to anxiety and job burnout and are usually driven to experience psychiatric disorders [21]. Religiosity and religion, in this context, have been linked to beneficial outcomes in individuals’ lives, including enhanced self‐esteem, improved quality of life, and greater psychological well‐being [21]. In a study among Iranian nurses, positive religious coping was associated with high quality of work life [22]. A separate study conducted among nursing students in Bosnia and Herzegovina suggested that higher levels of religiosity might serve as a potential resilience factor within this specific population [23]. A study conducted in Uganda demonstrated that religion plays a role in shaping nurses’ professional lives and coping strategies. It also found that religious values influence job performance and that engaging in various religious practices supports nurses in managing work‐related stress [24]. This indicates that religious beliefs not only support individual resilience but also shape workplace interactions, contributing to a culture of cooperation and moral responsibility in challenging clinical settings.

On the other hand, studies show that negative perceptions of God and religion can lead to poor psychological well‐being and distress [25]. In general, nurses’ religiosity is a fundamental resource that increases their adaptive potential, enables them to gain a “sense of support,” and defines the purpose and meaning of any endeavor. The frequency of manifestations of religiosity in everyday life and the degree of personal religiosity positively impact their personal resilience [21]. These results further confirm the consistency of our findings with the broader literature. In parallel with these findings, the metaphors such as “guide,” “compass,” “armor,” “savior,” “root,” and “worship” used by the nurses within the scope of the research concretely reveal the meaning and function of religious beliefs in their professional lives. These metaphors suggest that religious belief contributes to nurses’ orientation in stressful and challenging work environments (“guide” and “compass”), psychological protection (“armor”), access to sources of strength and resilience (“savior” and “root”), and integration of their professional practices with a spiritual meaning (“worship”).

In the study, the nurses stated that religious beliefs provide a positive clinical environment by providing a happy, harmonious/supportive working environment, increasing productivity/motivation, providing tolerance, ensuring patient/employee satisfaction, and encouraging patient care. This finding shows that nurses’ religious beliefs positively affect the work environment. However, it is quite meaningful that the nurses described the positive clinical environment using metaphors such as “spring morning,” “amusement park,” “spring weather,” “sea,” “relief,” and “peace.” These metaphors envision a work environment filled with positive emotions such as relaxation, refreshment, happiness, and vitality. This shows that the inner strength and values nurses derive from their beliefs have an impact not only on their moods but also on the general atmosphere around them. Similarly, it is stated in the literature that religious beliefs increase individuals’ job satisfaction, motivation, and tolerance levels, thus creating a more harmonious and supportive working environment [26, 27]. From a managerial perspective, these insights underline the importance of healthcare organizations and nurse leaders recognizing and respecting nurses’ value systems. By integrating ethical and cultural sensitivities into team communication and professional development programs, institutions can foster supportive and inclusive work environments that enhance motivation, collaboration, and patient‐care quality. Religion in this context significantly impacts people’s behavior and allows them to interact with different aspects of life. At the same time, a person’s spirituality and psychology significantly affect personal development [27]. Religious beliefs can also help individuals cope with stress and overcome challenges. Moreover, religion appears to support reducing mental pressures and offense and increasing life satisfaction [26].

The findings of this study reflect the context of eastern Turkey, where religious traditions strongly influence both daily life and professional practice. For this reason, the positive role of religion in creating supportive work environments may be specific to this setting. When considering transferability, caution is needed in applying these results to Western or more secular health systems, where religious beliefs may play a different or less central role in clinical practice.

Certain limitations should be considered when interpreting these findings. Conducting the study in a single hospital in eastern Turkey may restrict transferability, and as a qualitative design, the results are not intended for generalization. Even so, these factors do not lessen the value of the insights gained into how nurses’ religious beliefs relate to positive clinical work environments.

## 5. Conclusion

According to the study, nurses’ religious beliefs significantly influenced their professional and team relationships. Participants highlighted that these beliefs strengthened patience, ethical responsibility, respect, fairness, positive perspective, stress management, cooperation, and empathy. They also emphasized the supportive role of religious beliefs in coping with clinical difficulties, remaining calm, trusting, and acting in line with moral principles. Furthermore, religious beliefs were seen to contribute to a positive clinical environment by fostering harmony, motivation, tolerance, satisfaction, and quality care.

In this context, creating work environments that respect employees’ individual values and belief systems should be encouraged. To achieve this.

• Seminars, workshops, and spiritual support services can be provided.
• Professional training programs may include ethics, empathy, patience, and stress management, while considering religious and cultural sensitivities.
• Team communication training that promotes tolerance, understanding, and cooperation can be organized.
• Nurse managers should adopt leadership approaches sensitive to the religious and spiritual needs of staff to increase motivation and improve care quality.

## Disclosure

All listed authors meet the authorship criteria, and all authors agree with the content of the manuscript.

## Conflicts of Interest

The authors declare no conflicts of interest.

## Author Contributions

SÖ: conceptualization, methodology, investigation, writing–original draft, writing–review and editing, and supervision. GBT: conceptualization, methodology, investigation, conceptualization investigation, writing–original draft, and writing.

## Funding

This article was invited and accepted for publication by the journal; no publication fees were paid.

## Data Availability

The data supporting this study’s findings are available from the corresponding author upon reasonable request.
